# EFL learners’ motivation and acceptance of using large language models in English academic writing: an extension of the UTAUT model

**DOI:** 10.3389/fpsyg.2024.1514545

**Published:** 2025-01-20

**Authors:** Qingran Wang

**Affiliations:** School of Foreign Studies, China University of Political Science and Law, Haidian, Beijing, China

**Keywords:** large language models (LLMs), academic writing, motivation, unified theory of acceptance and use of technology (UTAUT), EFL courses

## Abstract

Large language models (LLMs), represented by ChatGPT, are one of the most significant technological breakthroughs in generative AI and have begun to be applied in EFL writing instruction. The advent of LLMs presents both opportunities and challenges for EFL learners, underscoring the importance of empirical evidence on their motivation and acceptance of using LLMs in learning English academic writing. This study recruited 238 participants who had completed one semester of training in using LLMs for business-related English academic writing. Participants answered question items based on the L2 Motivational Self System and the Unified Theory of Acceptance and Use of Technology (UTAUT). Partial least squares structural equation modeling (PLS-SEM) was employed to examine the structural relationships between the variables of motivation, region, previous learning experience, and the UTAUT model. Additionally, the moderating effect of motivation on the relationship between the four UTAUT determinants, behavioral intention, and use behavior was tested. The results show that performance expectancy and social influence significantly affect learners’ behavioral intention to use LLMs. Moreover, motivation proved to be a key factor in shaping both behavioral intention and actual use behavior, highlighting its crucial role in the adoption of technology for learning English academic writing.

## 1 Introduction

The integration of advanced technologies in education has significantly transformed how students approach learning, particularly in fields like business-related English academic writing (Xu and Wang, 2024a). One notable development is the advent of large language models (LLMs) such as OpenAI’s GPT series, which have shown immense potential in assisting learners by generating coherent text, providing real-time feedback, and improving the overall quality of written work (Liu and Ma, 2024; Su et al., 2023; Lu et al., 2024). At the same time, accelerated urbanization in China has fueled rapid economic growth but also exacerbated educational disparities between urban and rural areas (Zhang, 2017). This divide is evident in the varying English proficiency levels of English as a Foreign Language (EFL) learners in Chinese universities (Murray et al., 2023). To address these disparities, UNESCO advocates for the use of artificial intelligence (AI) to promote educational equity by catering to diverse learner needs and fostering inclusive education (Holmes and Miao, 2023). However, while the application of LLMs in English as a Foreign Language (EFL) learning contexts holds great promise, the factors influencing the acceptance and adoption of these tools by Chinese rural and urban EFL learners in higher education, particularly in academic writing, remain underexplored.

This study addresses this gap by investigating the factors influencing the adoption of LLMs for learning business-related English academic writing among Chinese rural and urban EFL learners. These factors include motivation, regional background, previous learning experience, performance expectancy, effort expectancy, social influence, and facilitating condition, with the Unified Theory of Acceptance and Use of Technology (UTAUT) model proposed by Venkatesh et al. (2003) serving as the theoretical foundation. The UTAUT model is the leading framework used by academia to explore users’ acceptance of technology (Wu et al., 2019), considering performance expectancy, effort expectancy, social influence, and facilitating conditions as key predictors of technology acceptance. These variables are particularly useful in understanding how students perceive and utilize digital tools for EFL learning (Guggemos et al., 2020; Hsu, 2023; Menon and Shilpa, 2023; Grassini et al., 2024). To expand the model’s explanatory power, motivation is introduced as both a predictor and a moderating variable, while students’ region and previous learning experiences (including whether they have ever taken computer courses and their streams in high school between science and arts) are also included as predictor variables.

The research was conducted at a prestigious university in Beijing, involving 238 undergraduate students enrolled in *Business English Writing* course. The course aimed to enhance students’ academic writing skills in business studies by using LLMs for tasks such as register analysis, lexico-grammatical analysis, paraphrasing, text evaluation, generating written corrective feedback, as well as data analysis and reporting. A survey was administered to assess learners’ motivation, performance expectancy, effort expectancy, social influence, facilitating condition, behavioral intention, and behavior use concerning LLMs adoption, and the data were analyzed using Partial Least Squares Structural Equation Modeling (PLS-SEM).

Based on the UTAUT theory, this study proposes an analytical framework for examining the factors influencing Chinese EFL learners’ acceptance of LLMs in business academic writing. This theoretical framework is applicable for a comprehensive and objective investigation of the impact of new technologies on student agency in technology-enhanced language learning courses and can serve as a reference for subsequent researchers conducting further analyses in related fields. Additionally, unlike business English courses in other Asian countries, business English courses in Chinese universities tend to be larger in size, with class sizes typically ranging from 40 to 50 students (Wang and Xu, 2023). Moreover, within the same classroom, students come from both underdeveloped regions (such as county-level cities or rural areas) and developed regions (such as municipalities or prefecture-level cities). The significant wealth gap between students’ families may lead to considerable differences in their motivations for learning English and their acceptance of new technologies. Furthermore, their prior exposure to computer courses during high school, along with the distinction between arts and science streams, may further amplify these differences. Therefore, this study can provide empirical insights for large EFL writing courses with significant background diversity. This study is structured into six sections: Section 1 introduces the research background, significance, and both theoretical and practical implications; Section 2 reviews the literature on the UTAUT model, motivation, and LLMs; Section 3 presents the hypotheses of the study; Section 4 describes the data and research methods; Section 5 analyzes and discusses the statistical results; and Section 6 concludes the study.

## 2 Literature review

### 2.1 The UTAUT model and its application in technology-enhanced writing courses

The UTAUT model is a comprehensive framework that integrates elements from the Technology Acceptance Model (TAM), developed by Fred Davis (1989), and seven other models: the Theory of Reasoned Action, the Motivational Model, the Theory of Planned Behavior, a combined Theory of Planned Behavior/Technology Acceptance Model, the Model of Personal Computer Use, the Diffusion of Innovations Theory, and Social Cognitive Theory (Venkatesh et al., 2003). The UTAUT model aims to predict user acceptance of technology by considering various factors and individual differences, including four core variables: performance expectation, effort expectation, social influence, and facilitating conditions, along with moderating variables that affect these core variables. The constructs of the UTAUT model offer valuable insights into how these factors influence technology adoption in educational settings, particularly in the technology-enhanced writing courses.

One significant application of the UTAUT model in writing courses is its role in predicting students’ acceptance of digital writing tools. Research indicates that performance expectancy significantly influences students’ intentions to use writing software (Zeng, 2019). For instance, a study by Grassini et al. (2024) revealed that students who believed using technology would enhance their writing skills were more likely to engage with generative AI tools. This suggests that educators can foster technology acceptance by highlighting the benefits of writing tools in improving students’ academic performance. Effort expectancy, which refers to the perceived ease of use, is another crucial factor in the adoption of technology in writing courses (Guggemos et al., 2020). Studies have shown that when students find digital tools intuitive and user-friendly, their willingness to utilize these resources increases (Budhathoki et al., 2024). This aligns with findings by Ferdousi (2022), who noted that simplifying the interface of writing software led to higher student engagement and satisfaction. Therefore, it is essential for educators to select and implement tools that minimize complexity to encourage their use in writing instruction. Social influence, which reflects the impact of peers and instructors on technology adoption, also plays a vital role in writing courses (Guggemos et al., 2020). This suggests that educators’ attitudes towards technology can significantly affect students’ perceptions and willingness to engage with digital writing resources. By actively promoting the integration of technology in writing courses, educators can create a supportive environment that fosters student engagement. Facilitating conditions, encompassing the resources and support available for technology use, are critical in the UTAUT framework as well (Hsu, 2023). Studies have shown that adequate access to technological resources, such as computers and internet connectivity, directly affects students’ ability to engage with digital writing tools (Menon and Shilpa, 2023). This support is particularly vital in diverse classrooms where students may have varying levels of technological proficiency.

Moreover, the UTAUT model’s emphasis on behavioral intention and use behavior allows for a nuanced understanding of how students interact with technology in writing contexts. Lin and Lai (2019) suggested that the limited ability of behavioral intention to predict actual use of educational technology might be due to the moderating influence of users’ self-regulation, with motivation being a key element of this self-regulation (Baumeister et al., 2007). This relationship underscores the importance of addressing motivational factors in technology adoption, as motivated students are more likely to integrate digital tools into their writing processes.

In conclusion, the application of the UTAUT model in technology-enhanced writing courses provides valuable insights into the factors influencing technology acceptance. By focusing on constructs such as performance expectancy, effort expectancy, social influence, and facilitating conditions, educators can better understand and facilitate students’ engagement with digital writing tools. However, Menon and Shilpa (2023) suggested that the predictive power of the UTAUT variables is influenced by context. Thus, exploring its application in the context of LLMs is important. This study utilizes the UTAUT model to gain a better understanding of EFL learners’ acceptance of LLMs for learning business-related English academic writing.

### 2.2 Motivation as a predictor and moderator in the UTAUT model

Educational disciplines have highlighted the critical role of learners’ motivation, as it directly affects their academic performance, facilitates the transfer of acquired knowledge, and reinforces their persistence in learning (Stroet et al., 2015). Moreover, motivation plays a key role in language acquisition, being closely associated with learners’ attitudes toward language learning and significantly influencing their efforts (Gardner, 2006; Wu, 2022). Research also indicates that motivation directly impacts users’ intentions to adopt technology in educational settings. For example, Davis et al. (1992) suggested that intrinsic motivation significantly shapes users’ perceptions of usefulness and ease of use—core components of the UTAUT framework. This finding is supported by Hsu (2023), who found that students with higher intrinsic motivation were more likely to perceive educational technologies as beneficial, thereby increasing their behavioral intention to use them.

Rafiee and Abbasian-Naghneh (2021) emphasized that learners’ motivation should be included as a key factor in any research model within the field of education. Additionally, motivation has been recognized as a critical variable in explaining individuals’ use of information technology (Roca and Gagné, 2008; Liu et al., 2024). Beyond serving as a predictor, motivation also acts as a moderator within the UTAUT model. For instance, Hsu (2023) found that motivation strengthens the relationship between social influence and EFL learners’ intention to use LMOOCs. When learners are motivated, they are more responsive to social recommendations about technology use, suggesting that motivation amplifies the effects of the social influence construct in the UTAUT framework.

Based on the above research, understanding the role of motivation enhances insights into user intention and behavior regarding technology adoption in educational settings. Therefore, we incorporate motivation as both a predictor and a moderating variable within the UTAUT model. This approach measures the impact of motivation on EFL learners’ intention and behavior in using LLMs for learning business-related English academic writing, as well as its moderating effects within the model.

### 2.3 LLMs and their application in teaching and learning academic writing

The advent of LLMs, such as OpenAI’s GPT series and ERNIE Bot, has transformed the educational landscape, particularly in the teaching and learning of academic writing. These models harness vast amounts of text data to generate coherent and contextually relevant content, thereby providing valuable tools for students and educators alike. One of the most significant advantages of using LLMs in teaching and learning academic writing is their ability to offer immediate feedback. Studies have shown that real-time feedback can enhance students’ academic writing skills by providing insights into the content, organization, vocabulary, grammar, syntax, cohesion, and mechanics of their writing (Xu and Wang, 2024a). For instance, LLMs can complement teacher assessment, which fosters a more iterative writing process (Lu et al., 2024). This interactive approach encourages students to engage with their writing actively, ultimately leading to better outcomes (Su et al., 2023).

Moreover, LLMs can serve as personalized writing assistants, catering to individual learning needs. Research indicates that ChatGPT is a valuable tool for learners at various proficiency levels to receive effective internal feedback (Tam, 2024). By adapting to the user’s writing style and providing specific suggestions, LLMs can help students develop their unique voices while adhering to academic conventions (Xu and Wang, 2024b). This personalization is particularly beneficial in diverse classrooms where students may have varying levels of experience and expertise in writing. In addition, LLMs can enhance the writing process by assisting students in generating ideas and structuring their arguments. By querying an LLM, students can receive suggestions for relevant topics, thesis statements, arguments, counterarguments, and even outlines for their papers. This capability is especially useful for novice writers who may struggle with the initial stages of the academic writing process (Su et al., 2023). By streamlining the brainstorming phase, LLMs can reduce writing anxiety, allowing students to focus more on content development.

However, the use of LLMs in teaching and learning academic writing is not without challenges. Concerns have been raised about the potential for academic dishonesty (Rudolph et al., 2023; van Dis et al., 2023), particularly as students might over-rely on AI-generated content, thereby undermining their critical and creative thinking during the writing process (Barrot, 2023). To mitigate this risk, educators must emphasize the importance of critical thinking and originality in writing. Integrating LLMs into the curriculum should involve discussions about ethical use and the role of these tools as aids rather than crutches. Furthermore, while LLMs are powerful, they are not infallible. Issues such as biases in training data and the hallucination problems necessitate careful consideration and oversight in their application (OpenAI, 2022; Thorp, 2023; Barrot, 2023; Xu and Wang, 2024b). Educators must remain vigilant and guide students in critically evaluating the outputs of LLMs, fostering a mindset of discernment in the use of technological tools.

In conclusion, the application of LLMs in teaching and learning academic writing presents both opportunities and challenges. While previous studies have employed the TAM model to explore EFL learners’ acceptance of LLMs in informal digital learning of English (Liu and Ma, 2024), to the best of our knowledge, there is limited research that has utilized the UTAUT model to analyze EFL learners’ acceptance of LLMs in learning business-related English academic writing, as well as the direct and moderating effects of motivation within the UTAUT model.

## 3 Hypotheses

### 3.1 Performance expectancy

Performance expectancy is the extent to which an individual believes that using a specific technology enhances their ability to perform tasks or achieve objectives (Venkatesh et al., 2003). It influences a person’s intention to adopt that technology (Kumar and Bervell, 2019). In academic settings, performance expectancy plays a crucial role in determining the adoption of a particular technology (Budhathoki et al., 2024). In this research, performance expectancy refers to the belief among Chinese university students that using LLMs will improve their academic writing skills in business studies. Based on this, we hypothesize:

RH1: EFL learners’ performance expectancy towards using LLMs would significantly affect their behavioral intention to use it.

### 3.2 Effort expectancy

Effort expectancy refers to the extent to which an individual perceives a particular technology as easy to use and requiring minimal effort (Venkatesh et al., 2003). It influences a person’s intention to adopt that technology (Guggemos et al., 2020). In this study, effort expectancy represents the belief among students in Chinese higher education that LLMs are easy to use and require little effort. Based on this, we hypothesize:

RH2: EFL learners’ effort expectancy towards using LLMs would significantly affect their behavioral intention to use it.

### 3.3 Social influence

Social influence refers to the extent to which an individual feels that important people in their life believe they should use a particular technology (Venkatesh et al., 2003). In this study, social influence pertains to how much students in Chinese universities believe that their peers, teachers, and other influential figures in their social network encourage them to use LLMs. Based on this, we hypothesize:

RH3: EFL learners’ social influence towards using LLMs would significantly affect their behavioral intention to use it.

### 3.4 Facilitating conditions

Facilitating conditions refer to the extent to which individuals believe they have the necessary resources and support to use a particular technology effectively (Venkatesh et al., 2003). Studies have shown that facilitating conditions influence students’ intentions to adopt new technologies (Kumar and Bervell, 2019; Hsu, 2023). In this study, facilitating conditions refer to how much students believe they have sufficient access to LLMs, along with the knowledge and resources required to use them effectively in their learning. Based on this, we hypothesize:

RH4: EFL learners’ facilitating conditions towards using LLMs significantly affect their use behavior.

### 3.5 Behavioral intention

Previous research indicates that behavioral intention has a positive, direct, and significant effect on technology usage behavior (Šumak and Šorgo, 2016; Budhathoki et al., 2024). This study suggests that performance expectancy, effort expectancy, and social influence directly and positively influence behavioral intention, which in turn positively affects usage behavior. Based on this, we hypothesize:

RH5: EFL learners’ behavioral intention towards using LLMs would significantly affect their use behavior.

### 3.6 Motivation

Rafiee and Abbasian-Naghneh (2021) highlighted the importance of including learners’ motivation as a key factor in any research model in the field of education. Moreover, motivation has been identified as a crucial factor in understanding individuals’ use of information technology (Roca and Gagné, 2008; Liu et al., 2024). In addition to being a predictor, motivation also functions as a moderator within the UTAUT model (Hsu, 2023). Based on this, we hypothesize:

RH6: EFL learners’ motivation significantly affects their behavioral intention towards using LLMs.

RH7: EFL learners’ motivation significantly affects their LLMs use behavior.

RH8: EFL learners’ motivation has a significant moderating effect on the relationship between the variables of UTAUT in LLMs settings.

### 3.7 Other factors

Due to the differences between business English courses in Chinese higher education and those in other countries and regions—particularly in terms of large class sizes and significant variations in both students’ family economic backgrounds and previous learning experiences in high school (Wang and Xu, 2023)—we expanded the UTAUT model by including variables such as students’ region (whether they come from developed areas like municipalities or prefecture-level cities, or less developed areas like county-level cities or rural regions), as well as their previous learning experiences (such as whether they have taken computer courses and their high school focus on science or arts), as predictor variables. Based on this, we hypothesize:

RH9: EFL learners’ regional background significantly affects either their behavioral intention or use behavior regarding LLMs.

RH10: EFL learners’ computer experience significantly affects either their behavioral intention or use behavior regarding LLMs.

RH11: EFL learners’ high school stream significantly affects either their behavioral intention or use behavior regarding LLMs.

## 4 Data and methods

This study recruited 238 first-year undergraduate students from the School of Business at a prestigious university in Beijing to participate in a survey regarding their motivation and acceptance of LLMs in the *Business English Writing* course. The course aims to enhance EFL learners’ English academic writing skills in the field of business through nine dimensions of assessment (vocabulary, grammar, orthographical control, genre format, cohesion & coherence, strategic competence, sociolinguistic competence, intercultural competence, and business knowledge) proposed by Wang and Fan (2020). In the course, we introduced participants to various LLMs to demonstrate their application methods and effects in business-related English academic writing, asking students to utilize them in their academic writing practice. Table 1 provides information about the writing module, teaching objectives, teaching methods, and the LLMs used.

**Table 1:** LLMs-assisted instruction in the *Business English Writing* course.

Module	Teaching objectives	Teaching method	LLMs^1^
Analysis of texts on the same business topic across different language registers	Develop students’ register awareness in English academic writing within the field of business, and enhance their understanding of the differences in word and sentence styles between formal and informal English.	Use LLMs to generate two essays on the same business topic, one in a formal style and one in an informal style. Arrange group discussions for students to summarize the similarities and differences between the two essays. Additionally, compare these essays with their own writings to reflect on issues related to the language registers of their essays.	ERNIE Bot; ChatGLM; Kimi
Analysis of words, phrases and expressions related to specific business topics and case report writing	Enable students to master keywords and phrases related to specific business topics, as well as commonly used expressions related to case report writing. Help students express their viewpoints more clearly and logically in English academic writing within the field of business.	Based on the writing topic and type, design different prompts to guide LLMs in generating commonly used words, phrases, and expressions related to specific business topics and case report writing.	ERNIE Bot; ChatGLM; Kimi
Paraphrase	Encourage students to use newly acquired English vocabulary, phrases, and common expressions to paraphrase existing simple paragraphs. By expanding and deepening the existing information in the paraphrase exercise, help students better master English academic writing in the field of business.	Design different prompts based on teaching tasks to make LLMs generate commonly used vocabulary related to relevant business topics, as well as original and paraphrased paragraphs for practice.	ERNIE Bot; ChatGLM; Kimi
Evaluating text & providing written corrective feedback	Guide students in using LLMs for self-assessment and to receive detailed written corrective feedback. Help students more precisely identify areas for improvement and reflect on their writing.	By guiding students in designing different prompts, encourage them to use LLMs to evaluate their writing and obtain written corrective feedback on aspects such as vocabulary, grammar, spelling, structure, coherence, and originality.	ERNIE Bot; ChatGLM; Kimi
Data Analysis and Reporting	Guide students in using LLMs to analyze relevant data and present statistical results while writing case reports.	By guiding students in designing different prompts, encourage them to use LLMs to extract data from text, reform data, classify and score text, extract sentiment, etc.	ERNIE Bot; ChatGLM; Kimi
Wrap-up	By systematically reviewing the above teaching modules, students will gain a better understanding of the content they have learned.	By combining the activities of writing exercises, peer feedback, and teacher feedback, enhance students’ ability to use LLMs in business English academic writing.	ERNIE Bot; ChatGLM; Kimi

^1We have incorporated three LLMs—ERNIE Bot, ChatGLM, and Kimi—into classroom teaching. Developed by leading Chinese high-tech companies, these LLMs represent the cutting edge of LLM development in China.^

In this study, we used SurveyMonkey to administer a questionnaire to 242 students enrolled in the *Business English Writing* course to investigate their motivation and acceptance of LLMs for learning English academic writing in the field of business, as well as their regional backgrounds and prior learning experiences. In the questionnaire, we explained the purpose of the study to the students, informing them that participation was completely voluntary and that they could withdraw at any time. Not participating or withdrawing would not affect their course grades. Ultimately, four students opted out of the survey, resulting in 238 valid responses. Throughout the research process, participants’ identities were kept confidential.

**Figure 1:**
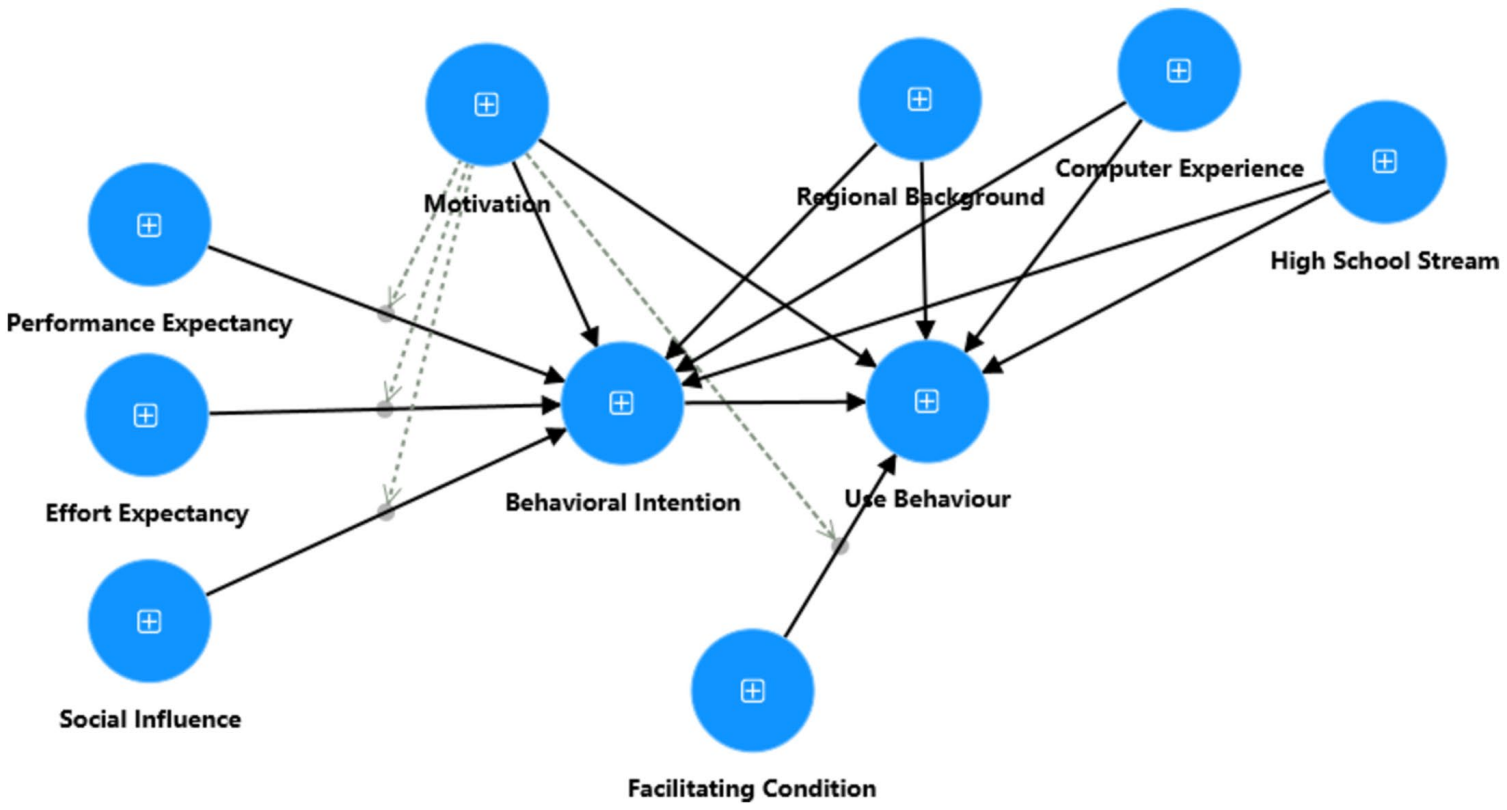
The proposed research model.

This study used the key motivational factors proposed by Taguchi et al. (2009) to measure EFL learners’ motivation toward LLMs in learning business-related English academic writing. These factors include integrativeness, instrumentality, attitudes, and two criterion measures, namely language choice preference and the learners’ intended learning effort. Based on previous research (Venkatesh et al., 2003; Hsu, 2023), we incorporated motivation as a key latent variable into the UTAUT model and proposed the theoretical model for this study, as shown in Figure 1. This model proposes that behavioral intention serves as a crucial mediator between constructs such as motivation, performance expectancy, effort expectancy, social influence, and use behavior. Additionally, motivation, facilitating conditions, regional background, computer learning experience, and high school stream directly influence the use behavior, indicating that both psychological factors and external conditions play a key role in technology adoption. PLS-SEM was applied as the statistical technique to explore the structural relationships among variables. Furthermore, motivation was used as a moderating variable in the relationships between performance expectancy and behavioral intention, effort expectancy and behavioral intention, social influence and behavioral intention, as well as facilitating conditions and use behavior.

### 4.1 Participants

We conducted this survey in the *Business English Writing* class at a prestigious university in Beijing, involving students from four business majors: finance, economics, business administration, and international business. A total of about 238 students participated in the experiment. Of the participants, roughly 60% came from developed regions of China, while around 40% were from underdeveloped regions. Additionally, around 16% had taken computer courses in high school, while the remaining 84% had not. Regarding their high school streams, about 25% were in the art stream, around 59% were in the science stream, and approximately 16% did not differentiate between the arts and science streams. All students participated voluntarily and were assured that the survey results would be used solely for academic research. All students participated voluntarily and were assured that the survey results would be used solely for academic research.

### 4.2 Measures

This study applied the L2 Motivational Self-System proposed by Taguchi et al. (2009) and the UTAUT model developed by Venkatesh et al. (2003) to measure Chinese EFL learners’ motivation and acceptance of using LLMs in English academic writing for business studies. To better fit the scope of this research, we revised the motivation-related question items based on the English Learning Motivation Questionnaire (ELMQ) developed by Taguchi et al. (2009). Additionally, the UTAUT items were developed following studies by Venkatesh et al. (2003) and Hsu (2023). Specifically, 9 items assessed participants’ motivation (e.g., ‘Even if it’s not required, I’m willing to use LLMs to learn business-related English academic writing.’). In the UTAUT model, five items evaluated participants’ performance expectancy of LLMs (e.g., ‘I find LLMs to be useful for learning business-related English academic writing.’), and three items examined their effort expectancy (e.g., ‘I find LLMs for learning business-related English academic writing flexible and easy to use.’). Three items measured participants’ social influence regarding LLM use (e.g., ‘I would use LLMs for learning business-related English academic writing if my peers recommended it to me.’), and three items assessed the facilitating conditions (e.g., ‘There is adequate training on the use of LLMs for learning business-related English academic writing in my university.’). Finally, participants’ behavioral intention was measured with two items (e.g., ‘I plan to use LLMs for learning business-related English academic writing.’), and behavior use was assessed with two items (e.g., ‘I would enjoy using LLMs for learning business-related English academic writing.’).

The questionnaire utilized a 6-point Likert scale, which included the categories: strongly agree, agree, slightly agree, slightly disagree, disagree, and strongly disagree. The composite reliability for each construct was above 0.7, confirming the reliability of the items (Fornell and Larcker, 1981a). To ensure that students understood the questionnaire accurately and to guarantee its validity, we first translated the questionnaire into Chinese and then back-translated it into English. Finally, we distributed the Chinese version of the questionnaire to participants via SurveyMonkey.

### 4.3 Data analysis

This study used Partial Least Squares Structural Equation Modeling (PLS-SEM) to conduct structural equation analysis. According to Hair et al. (2021), the advantages of PLS-SEM are as follows: (1) PLS-SEM can handle small sample sizes and non-normal data distributions effectively, making it suitable for exploratory research where large samples may not be feasible; (2) PLS-SEM emphasizes maximizing explained variance in the dependent constructs, which is beneficial for predictive modeling and understanding relationships in complex models; (3) PLS-SEM can effectively model both reflective and formative constructs, allowing for a more nuanced understanding of how different types of variables interact; (4) PLS-SEM is less sensitive to multicollinearity issues compared to traditional covariance-based SEM methods, making it a good choice when predictors are highly correlated; and (5) The output from PLS-SEM is relatively straightforward, enabling researchers to easily interpret path coefficients and their significance, which aids in communicating findings.

Additionally, PLS-SEM is particularly useful for examining developing theories due to its higher statistical power (Hair et al., 2019). Therefore, this study adopted SmartPLS 4.0 for PLS-SEM analysis. The specific analysis steps were as follows: First, we assessed the overall reliability of the proposed model using R^2^ and evaluated the reliability of the constructs using composite reliability (CR). Next, we employed the PLS-SEM algorithm in the software to estimate the path coefficients between variables and the moderating effect of motivation. Finally, we used bootstrapping to evaluate the confidence intervals and significance levels of the path coefficients, determining whether the path coefficients in the model were significant and providing the relevant *p*-values.

To assess the potential presence of common method bias (CMB) in our data, we employed Harman’s single-factor technique (HSF), a widely used method for detecting CMB issues. The results of the exploratory factor analysis (EFA) showed that the first factor accounted for 38.53% of the total variance in the dataset. According to the guidelines in the literature, a “total variance explained” by the first component (or factor) extracted below 50% typically suggests that common method bias is not a serious concern (Podsakoff et al., 2003; Kock, 2020). Therefore, based on this result, we conclude that the dataset does not exhibit significant common method bias.

## 5 Results and discussion

Before analyzing the PLS-SEM results, it is crucial to verify the reliability and validity of the proposed research model. Table 2 provides the model and construct fit measures for the proposed model, focusing on R^2^ and SRMR values to evaluate the model’s predictive power, as well as Cronbach’s *α*, Composite Reliability (rho_c), and AVE to assess the reliability of the constructs. As shown in Table 2, The R^2^ values for behavior intention (0.55) and use behavior (0.55) are relatively high, meaning that the UTAUT model explains a substantial portion of the variance in these constructs (Hair et al., 2011). The Standardized Root Mean Residual (SRMR) value of the proposed model is 0.07, which indicates that the model’s predicted relationships closely match the observed data (Schermelleh-Engel et al., 2003). The R^2^ and SRMR values demonstrate that the UTAUT model provides a strong explanation for participants’ intentions to use and actual usage of LLMs in learning English academic writing. This suggests that key factors such as performance expectancy, effort expectancy, social influence, and motivation strongly predict both the likelihood of participants forming intentions to use LLMs and their actual usage.

**Table 2:** Model and construct fit.

Fit measures	Endogenous construct		
R^2^	BI = 0.55; UB = 0.55		
SRMR	0.07		
	Construct reliability and validity		
Construct	Cronbach α	CR (rho_c)	AVE
Motivation	0.87	0.90	0.51
Performance Expectancy	0.87	0.91	0.67
Effort Expectancy	0.81	0.88	0.72
Social Influence	0.81	0.88	0.66
Facilitating Condition	0.80	0.70	0.50
Behavioral Intention	0.71	0.87	0.77
Use Behavior	0.82	0.91	0.84

To assess convergent validity, Cronbach’s α, construct reliability (CR), and average variance extracted (AVE) were computed. A generally accepted rule is that a Cronbach’s α of 0.7–0.8 indicates an acceptable level of reliability, while 0.8–0.9 indicates an ideal level (Tavakol and Dennick, 2011). All of our constructs meet this criterion, which means that they exhibit good internal consistency. Furthermore, previous studies suggest that a CR of 0.6 or higher (Fornell and Larcker, 1981b) and an AVE of 0.5 or higher (Hair et al., 2010) are considered acceptable. As presented in Table 2, the AVEs for the seven latent constructs ranged from 0.50 to 0.84, meeting or exceeding the threshold, and the CR values for all constructs ranged from 0.70 to 0.91, exceeding the recommended value. These results confirm convergent validity and indicate good internal consistency for the constructs of the proposed model.

In addition, we calculate the Heterotrait-Monotrait ratio (HTMT) for motivation and UTAUT factors to assess discriminant validity between similar and different indicators. To establish discriminant validity, HTMT values must be lower than the threshold value of 0.90 (Gold et al., 2001; Teo et al., 2008). As shown in Table 3, all values are below 0.86. Furthermore, we calculated HTMT inference using bootstrapping with 5,000 subsamples. If the resulting interval is below 1, discriminant validity is confirmed (Henseler et al., 2015; Hernández-Perlines and Mancebo-Lozano, 2016). Our results also meet this criterion.

**Table 3:** Heterotrait---Monotrait ratio (HTMT).

	Original sample (O)	Sample mean (M)	2.5%	97.5%
Motivation ➡ Behavioral Intention	0.79	0.796	0.58	0.94
Motivation ➡ Effort Expectancy	0.47	0.483	0.29	0.66
Motivation ➡ Facilitating Condition	0.13	0.182	0.10	0.32
Performance Expectancy ➡ Behavioral Intention	0.85	0.856	0.73	0.96
Performance Expectancy ➡ Effort Expectancy	0.77	0.771	0.65	0.86
Performance Expectancy ➡ Facilitating Condition	0.11	0.167	0.08	0.31
Performance Expectancy ➡ Motivation	0.70	0.709	0.51	0.84
Effort Expectancy ➡ Behavioral Intention	0.63	0.630	0.46	0.77
Social Influence ➡ Behavioral Intention	0.83	0.830	0.70	0.93
Social Influence ➡ Effort Expectancy	0.70	0.702	0.54	0.82
Social Influence ➡ Facilitating Condition	0.15	0.191	0.07	0.40
Social Influence ➡ Motivation	0.60	0.612	0.41	0.77
Social Influence ➡ Performance Expectancy	0.80	0.799	0.68	0.88
Facilitating Condition ➡ Behavioral Intention	0.03	0.121	0.03	0.28
Facilitating Condition ➡ Effort Expectancy	0.21	0.236	0.09	0.42
Use Behaviour ➡ Behavioral Intention	0.86	0.867	0.76	0.95
Use Behaviour ➡ Effort Expectancy	0.59	0.594	0.39	0.76
Use Behaviour ➡ Facilitating Condition	0.08	0.137	0.05	0.26
Use Behaviour ➡ Motivation	0.74	0.742	0.56	0.87
Use Behaviour ➡ Performance Expectancy	0.75	0.748	0.63	0.84
Use Behaviour ➡ Social Influence	0.72	0.719	0.50	0.87

After confirming the fit of the proposed model, structural modeling was used to examine the relationships among the variables. As shown in Table 4, the findings of PLS-SEM reported that performance expectancy ( $\beta_{\mathrm{Performance}\;\mathrm{expectancy}}$ = 0.29, *p* = 0.002) and social influence ( $\beta_{\mathrm{Social}\;\mathrm{Influence}}$ = 0.22, *p* = 0.003) significantly influence EFL learners’ behavioral intention to use LLMs in business academic writing. However, effort expectancy ( $\beta_{\mathrm{Effort}\;\mathrm{Expectancy}}$ = 0.03, *p* = 0.588) was not statistically significant. Moreover, participants’ behavioral intention ( $\beta_{\mathrm{behavioral}\;\mathrm{intention}}$ = 0.48, *p* = 0.000) significantly influences their use behavior, while facilitating conditions ( $\beta_{\mathrm{facilitating}\;\mathrm{condition}}$ = −0.06, *p* = 0.388), regional background ( $\beta_{\mathrm{regional}\;\mathrm{background}}$ = 0.12, *p* = 0.153), computer experience ( $\beta_{\mathrm{computer}\;\mathrm{experience}}$ = −0.05, *p* = 0.692), and high school stream ( $\beta_{\mathrm{high}\;\mathrm{school}\;\mathrm{stream}}$ = −0.12, *p* = 0.417) were not significantly associated with use behavior.

**Table 4:** Path coefficients of PLS-SEM.

UTAUT	*β*	Standard deviation	*p*
Performance Expectance➡Behavior Intention	0.29***	0.09	0.002
Effort Expectancy➡Behavior Intention	0.03	0.06	0.588
Social Influence➡Behavior Intention	0.22***	0.07	0.003
Motivation➡Behavior Intention	0.29***	0.09	0.001
Regional Background➡Behavior Intention	0.12	0.08	0.153
Computer Experience➡Behavior Intention	−0.05	0.14	0.692
High School Stream➡Behavior Intention	−0.12	0.15	0.417
Facilitating Condition➡Use behavior	−0.06	0.07	0.388
Behavior Intention➡Use Behavior	0.48***	0.09	0.000
Motivation➡Use Behavior	0.33***	0.09	0.000
Regional Background➡Use behavior	−0.09	0.08	0.266
Computer Experience➡Use Behavior	0.04	0.10	0.645
High School Stream➡Use Behavior	0.23	0.25	0.340

*** represent the 1% significance level.

Our findings are partially in line with the studies by Guggemos et al. (2020). In their study, performance expectancy, effort expectancy, and social influence were strong predictors of users’ intention to use digital tools in academic writing classes. However, this study found that performance expectancy and social influence remain significant predictors of EFL learners’ intention to use LLMs in academic writing courses, while the variable of effort expectancy was not. The possible explanation for this discrepancy may be related to the career planning and development of undergraduate students in Chinese universities. For most undergraduate students majoring in business in China, obtaining a master’s degree or higher is required to secure better career development opportunities. Moreover, English academic writing ability is crucial for Chinese business undergraduates to qualify for a recommendation-based graduate admission or to perform well in the graduate entrance exams (Wang and Xu, 2023). Therefore, as long as students anticipate that LLMs can help improve their English writing skills, they are willing to use this generative AI tool in the learning process, regardless of the effort required.

In this study, performance expectancy significantly influences users’ behavioral intentions. This aligns with prior research that investigated students’ intention to adopt generative AI tools using the UTAUT model (Bin-Nashwan et al., 2023; Budhathoki et al., 2024). The result indicates that Chinese EFL learners are more likely to use LLMs if they believe these tools will improve their academic writing performance. Therefore, in the context of higher education in China, educators should emphasize the practical benefits of LLMs in enhancing writing quality and efficiency to foster adoption.

Social influence also has a notable impact, as confirmed by several studies (Maican et al., 2019; Guggemos et al., 2020; Budhathoki et al., 2024). The findings suggest that recommendations from peers and endorsements from instructors influence Chinese EFL learners’ intentions to use LLMs. Educators can leverage this by fostering a positive social atmosphere around LLM usage, encouraging peer discussions and collaborative work that support the adoption of these tools.

However, effort expectancy does not significantly affect behavioral intention in this study. This finding contradicts previous research (Guggemos et al., 2020; Kwak et al., 2022), and the possible reason, as mentioned above, may be related to the current career planning and development of undergraduate students in China. This study implies that learners’ perceptions of ease of use are not as critical in this context. Learners appear to be more focused on the anticipated performance gains rather than the effort required to use LLMs. As a result, Chinese educational institutions may not need to invest heavily in simplifying LLM interfaces or functionality, as perceived benefits and social influence are more influential factors.

Furthermore, behavioral intention is a strong predictor of actual LLM usage, aligning with previous studies that emphasize the importance of intention in technology adoption (Šumak and Šorgo, 2016; Budhathoki et al., 2024). This implies that once learners have a positive attitude toward using LLMs, they are highly likely to integrate them into their academic writing routines. Finally, factors such as facilitating conditions (e.g., external support systems), regional background, computer experience, and high school stream do not significantly influence usage, suggesting that once students form a positive intention, they are inclined to use LLMs regardless of other factors.

For the effect of participants’ motivation as a predictor of their behavioral intention and use behavior toward LLMs in learning business-related English academic writing, as presented in Table 4, results showed that motivation was a significant factor influencing both behavioral intention ( $\beta_{\mathrm{motivation}1}$ = 0.29, *p* = 0.001) and use behavior ( $\beta_{\mathrm{motivation}2}$ = 0.33, *p* = 0.000). Our findings are consistent with those of Wang and Zhan (2020) and Strzelecki (2023), which indicate that Chinese EFL learners’ motivation has a significant influence on both their intention to act and their actual behavior. The finding suggests that, beyond the traditional UTAUT constructs, motivation plays a crucial role in predicting how EFL learners engage with LLMs in learning business-related English academic writing. The inclusion of motivation aligns with extensions to the UTAUT model, emphasizing the importance of intrinsic factors like interest and desire in shaping EFL learners’ technology acceptance. Therefore, educators should focus on enhancing motivational elements such as autonomy, competence, and relatedness to encourage students’ adoption of LLMs in academic writing settings (Hsu, 2023).

This study also aims to explore the moderating effect of motivation on the variables of the UTAUT model. Table 5 shows the moderating effect of motivation on various variables within the UTAUT model, using *β* coefficients, standard deviations, and *p*-values to report the statistical significance of these relationships. The results indicated that motivation does not have a statistically significant influence on the relationships between the predictors and dependent variables under examination. Specifically, motivation does not significantly moderate the relationships between performance expectancy, effort expectancy, social influence, and behavioral intention. Similarly, for facilitating conditions, motivation does not significantly moderate its relationship with actual usage behavior.

**Table 5:** Moderating effect of motivation to variables of UTAUT.

UTAUT	*β*	Standard deviation	*p*
Motivation x Performance Expectancy ➡ Behavior Intention	−0.01	0.07	0.858
Motivation x Effort Expectancy ➡ Behavior Intention	0.03	0.06	0.616
Motivation x Social Influence ➡Behavior Intention	−0.04	0.06	0.499
Motivation x Facilitating Condition ➡ Use Behavior	0.03	0.08	0.674

The results suggest that motivation does not exhibit moderating effects on the UTAUT relationships, which may be because its influence on behavior could be less direct in the context of the model’s constructs. According to Venkatesh et al. (2003), UTAUT primarily emphasizes the role of performance expectancy, effort expectancy, social influence, and facilitating conditions in shaping behavioral intention and usage behavior. Motivation, while important in influencing individual intentions and actions, might not significantly alter how these factors directly influence technology adoption. This finding was partially echoed in Hsu (2023), which suggests that individual differences, like intrinsic motivation, might not always have a strong moderating effect in the face of more dominant predictors.

Table 6 provides a summary of the examination of research hypotheses, detailing whether each hypothesis was supported or not. To sum up, performance expectancy, social influence, and motivation are significant predictors of EFL learners’ behavioral intentions toward using LLMs in learning business-related English academic writing. Motivation and behavioral intention significantly influence EFL learners’ actual use of LLMs in learning academic writing. The effects of other variables are not significant.

**Table 6:** Summary of research hypotheses examination.

Research hypotheses	Results
RH1: EFL learners’ performance expectancy toward using LLMs would significantly affect their behavioral intention to it.	Supported
RH2: EFL learners’ effort expectancy toward using LLMs would significantly affect their behavioral intention to it.	Not Supported
RH3: EFL learners’ social influence toward using LLMs would significantly affect their behavioral intention to it.	Supported
RH4: EFL learners’ facilitating conditions using toward LLMs would significantly affect their use behavior.	Not Supported
RH5: EFL learners’ behavioral intention toward using LLMs would significantly affect their use behavior.	Supported
RH6: EFL learners’ motivation significantly affects their behavioral intention toward using LLMs.	Supported
RH7: EFL learners’ motivation significantly affects their LLMs use behavior.	Supported
RH8: EFL learners’ motivation has a significant moderating effect on the relationship between UTAUT variables in LLMs settings.	Not Supported
RH9: EFL learners’ regional background significantly affects either their behavioral intention or use behavior regarding LLMs.	Not Supported
RH10: EFL learners’ computer experience significantly affects either their behavioral intention or use behavior regarding LLMs.	Not Supported
RH11: EFL learners’ high school stream significantly affects either their behavioral intention or use behavior regarding LLMs.	Not Supported

Table 6 provides a summary of the examination of the research hypotheses, detailing whether each hypothesis was supported. To summarize, performance expectancy, social influence, and motivation are significant predictors of EFL learners’ behavioral intentions toward using LLMs in learning business-related English academic writing. Motivation and behavioral intention significantly influence EFL learners’ actual use of LLMs in academic writing. On the other hand, the effects of other variables are not significant.

## 6 Conclusion and contribution

This study investigated the factors influencing EFL learners’ adoption of LLMs in learning business-related English academic writing by applying the UTAUT model and incorporating motivation, regional background, and previous learning experiences (computer experience and high school streams) as key variables. The research primarily aimed to understand the effects of performance expectancy, effort expectancy, social influence, motivation, regional background, and previous learning experiences on both behavioral intention and actual usage of LLMs among EFL learners in academic writing contexts. Additionally, the study sought to explore the moderating effect of motivation on these relationships.

The major findings indicate that performance expectancy and social influence significantly predict EFL learners’ behavioral intention to use LLMs in their business-related English academic writing. Motivation also emerged as a significant predictor of both behavioral intention and use behavior, confirming its central role in encouraging the adoption of educational technologies in academic writing contexts. On the other hand, effort expectancy did not significantly influence behavioral intention, suggesting that learners might prioritize the perceived benefits of LLMs over the ease of using them. Interestingly, the facilitating conditions, which refer to the availability of resources and support for using LLMs, did not have a significant effect on learners’ actual use behavior. This could imply that, in the context of this study, learners already have adequate access to the resources needed to use LLMs, or that their motivation and the perceived benefits of the technology outweigh the importance of external support in learning academic writing. The moderating role of motivation was not supported by the empirical evidence, as motivation did not significantly alter the relationships between key UTAUT variables and behavioral intention. Moreover, although there are significant differences among students in terms of regional background and previous learning experience, these differences did not significantly impact their behavioral intention and use behavior.

This study contributes to the literature by providing both theoretical and practical implications for the use of cutting-edge technology in EFL writing courses. This study’s theoretical implications, grounded in UTAUT theory, offer an analytical framework to examine the factors that influence Chinese EFL learners’ acceptance of new technology in writing courses. This framework incorporates variables such as motivation, regional background, and differences in previous learning experience, which are common in EFL classrooms in Chinese higher education. It is highly applicable for a comprehensive and objective study of the agency of Chinese EFL learners in technology-assisted writing courses and can serve as a reference for researchers conducting further analysis in related fields.

Equally important, the findings also provide several practical implications for improving EFL learners’ acceptance of LLMs in learning academic writing, particularly in the field of business. First, interventions aimed at increasing performance expectancy and social influence should be prioritized, as these are key factors driving learners’ intention to use LLMs. Educators could emphasize the performance benefits of LLMs in enhancing writing skills or create a collaborative environment where peer encouragement fosters greater technology adoption. For instance, in the intervention, we asked students to work in groups to use LLMs to search for specific business terms (e.g., “digital transformation,” “sustainability,” “green finance,” “trade dispute/war”) and analyzed their frequency and usage patterns over a set period (e.g., the past five years). Next, students were required to create a timeline showing how the usage of each term has evolved, and reflect on why certain terms have gained or lost prominence based on business events or trends. Finally, students worked in groups to discuss and present the implications of these trends.

Through group collaboration, reporting, and reflection activities like these, students can gain insight into how their peers use LLMs to learn business English vocabulary and their reflections on its usage, thereby further enhancing their acceptance of this technology.

Second, motivation stands out as a crucial factor not only in shaping learners’ intention but also in driving actual usage. Therefore, fostering intrinsic motivation through engaging, relevant, and supportive learning environments is essential. Specifically, to increase EFL learners’ motivation toward using LLMs in business English academic writing, educators can adopt several effective strategies. First, goal-setting is essential; by providing clear, achievable objectives—such as improving writing quality or mastering key business terms—learners stay focused and motivated. Immediate feedback also plays a crucial role, allowing students to track their progress and adjust their approach accordingly, which reinforces their self-efficacy. In addition, educators can foster a supportive learning community by encouraging peer collaboration. Group activities, where students use LLMs to explore business-related vocabulary or analyze trends, can boost motivation through social interaction and shared learning experiences. Gamification elements, such as small rewards or recognition for achievements, can introduce an element of fun and competition, keeping learners engaged. Finally, real-world relevance is key. Educators should emphasize how LLMs can enhance learners’ business writing skills, showing them the direct impact on future academic and professional success, making the learning process more meaningful.

Our analysis, however, has limitations. First, the study focused solely on EFL learners in the context of academic writing in the field of business, which limits the generalizability of the findings to promoting the use of LLMs in academic writing in other fields. Second, although motivation was treated as a moderating variable, the results showed no support for its moderating effects, which may be due to the relatively small sample size. Finally, the study relied on self-reported data, which may be subject to biases such as social desirability or inaccurate self-assessment.

In the future, we plan to explore how these factors manifest across different academic fields and technologies to determine if the findings are consistent in varied educational settings. Additionally, we aim to increase the sample size and include more moderating variables, such as students’ learning style and metacognitive awareness, to gain a more comprehensive and robust understanding of the moderating effects of motivation and other factors within the UTAUT model. Future research could also incorporate more objective measures, such as actual usage data or performance assessments, to provide a clearer picture of how motivation and UTAUT factors influence EFL learners’ intentions and adoption of LLMs in academic writing contexts.

## Data Availability

The raw data supporting the conclusions of this article will be made available by the authors, without undue reservation.
