# Supplementary material for: EFL learners’ motivation and acceptance of using large language models in English academic writing: an extension of the UTAUT model
Source: Front Psychol. 2025 Jan 20;15:1514545. doi: 10.3389/fpsyg.2024.1514545 (PMC11788289; doi:10.3389/fpsyg.2024.1514545)
Supplement: Supplementary file 1 [file Data_Sheet_1.docx]

**Appendix A. Question items of motivation construct**

| Item |
| --- |
| 1. If I don’t use LLMs to learn business-related English academic writing, it will have a negative impact on my life. |
| 1. Using LLMs to learn business-related English academic writing is important to me in order to gain recognition from my peers/teachers/family/boss. |
| 1. If there are future courses that use LLMs to assist in learning business-related English academic writing, I would be willing to participate. |
| 1. Even if it’s not required, I’m willing to use LLMs to learn business-related English academic writing. |
| 1. Using LLMs to learn business-related English academic writing is important to me because if I have relevant knowledge, others will respect me more. |
| 1. My parents encourage me to use LLMs to business-related English academic writing in my free time. |
| 1. I can imagine myself using LLMs to write business-related English academic writing fluently. |
| 1. Using LLMs to learn business-related English academic writing is important to me because I plan to study abroad. |
| 1. I must learn business-related English academic writing because I cannot get my degree without passing Business English courses. |

**Appendix B. UTAUT Construct Questionnaire**

| UTAUT Construct | Item |
| --- | --- |
| Performance Expectancy | 1. I find LLMs to be useful for learning business-related English academic writing. |
|  | 1. Using LLMs could enable me to complete business-related English academic writing tasks more quickly. |
|  | 1. Using LLMs would increase my productivity in learning business-related English academic writing. |
|  | 1. Using LLMs for learning business-related English academic writing would improve my performance. |
|  | 1. Using LLMs for learning business-related English academic writing would enhance my confidence to use English for communication. |
| Effort Expectancy | 1. I find LLMs for learning business-related English academic writing flexible and easy to use. |
|  | 1. Learning to use LLMs for business-related English academic writing does not require much effort. |
|  | 1. It would be easy for me to become skillful at using LLMs for learning business-related English academic writing. |
| Social Influence | 1. I would use LLMs for learning business-related English academic writing if my peers recommended it to me. |
|  | 1. I would like to use LLMs for learning business-related English academic writing if my instructors supported the use of it. |
|  | 1. Instructors in my department have been helpful in the use LLMs for learning business-related English academic writing. |
| Facilitating Condition | 1. There is adequate training on the use of LLMs for learning business-related English academic writing in my university. |
|  | 1. The presence of unstable power supply hinders the effective use of LLMs for learning business-related English academic writing in my university |
|  | 1. Poor design of contents and technology support in my university do not motivate me to use LLMs for learning business-related English academic writing. |

| UTAUT Construct | Item |
| --- | --- |
| Behavioural Intention | 1. I plan to use LLMs for learning business-related English academic writing. |
|  | 1. I think I will use LLMs for learning business-related English academic writing frequently. |
| Use Behaviour | 1. I would enjoy using LLMs for learning business-related English academic writing. 2. I like to use LLMs for learning business-related English academic writing and I plant to use them more in the future. |
